# Deterrence or conformity?—An analysis of digital economy taxpayer compliance behavior based on prospect theory

**DOI:** 10.3389/fpsyg.2022.967715

**Published:** 2022-09-21

**Authors:** Peng Jin, Guiping Li, Weiqing Xiong

**Affiliations:** ^1^College of Finance and Information, Ningbo University of Finance and Economics, Ningbo, China; ^2^Business School, Ningbo University, Ningbo, China

**Keywords:** digital economy, taxpayer compliance, prospect theory, perceived compliance level, deterrence effect, conformity effect

## Abstract

Taking the tax evasion of live streamers as a reference case, this article aims to establish a tax compliance prospect theory model, in which the reference point is determined by the taxpayer's perceived compliance level. The taxpayer compliance behavior is analyzed from two dimensions of the tax authority's audit deterrence and the taxpayer's conformity mentality. We find that both deterrence effect and conformity effect are existing in the taxpayer compliance behavior, which is affected by the tax authority's enforcement effort. When the enforcement effort is low, the deterrence effect and the conformity effect do not exist, and the taxpayer does not comply at all. When the enforcement effort is moderate, the deterrence effect and the conformity effect coexist and the taxpayer complies partially. Besides, the taxpayer compliance level will not exceed the perceived compliance level, and an increase in the perceived compliance level will prompt the taxpayer to comply more. When the enforcement effort is high, there is only a strong deterrence effect, and the taxpayer completely complies. We use the theoretical analysis result to explain the tax compliance behavior of live streamers and find that it is consistent with the predictions of the model. Finally, we propose some policy recommendations on tax administration in digital economy.

## Introduction

Taxpayer compliance, also known as tax compliance, means that taxpayers fulfill their tax obligations in accordance with the law, otherwise it is called taxpayer non-compliance. Taxpayers' compliance or non-compliance can be subdivided into different types, such as selfish non-compliance, procedural non-compliance, ignorant non-compliance, and social non-compliance. Tax compliance research mostly focuses on investigating selfish non-compliance, which is “tax evasion.”

In 2017, when the phrase “digital economy” appeared in the Chinese government work report for the first time, and then the 2019 Chinese government work report continued to propose “strengthening the digital economy.” The following outbreak of COVID-19 in 2020 accelerated the digital transformation of the Chinese economy. According to the statistics from the China Academy of Information and Communications Technology, the amount of Chinese digital economy reaches 39.2 trillion yuan in 2020, accounting for 38.6% of gross domestic product (GDP) and with a year-on-year growth of 2.4 points, effectively supporting the epidemic control and the economy development. However, with the rapid development of the digital economy, the relevant tax problems are increasing correspondingly. The traditional tax legal system and the method of tax administration have not been yet fully adjusted to adapt to the business model of digital economy; therefore, many digital economy practitioners use the loopholes of tax legal system to evade taxes, resulting in huge tax losses. At present, the academia and the government have not yet calculated the amount of tax losses in Chinese digital economy, but according to Cai Chang, the director of the Tax Planning and Legal Research Center of the Central University of Finance and Economics, the tax losses caused by C2C e-commerce in 2018 exceeded 100 billion yuan.

Live streaming is seen as a new form of digital economy emerging in recent years, and its tax problems have attracted great attention from tax authorities. On November 22, 2021, the Hangzhou Municipal Taxation Bureau issued a punishment notice on the live streamers Zhu Chenhui (net name: Xueli Cherie) and Lin Shanshan (net name: Lin Shanshan_Sunny). They were charged with overdue taxes and fines of 65.5531 million yuan and 27.6725 million yuan, respectively. The method of these two live streamers' tax evasion is to convert individual wages, salaries, and labor incomes into the operating revenues of their sole proprietorship enterprises through fabricating business, in order to evade individual income tax. Then, on December 20, 2021, the Hangzhou Municipal Taxation Bureau found out that the famous live streamer Huang Wei (net name: Weiya) evaded taxes of 643 million yuan by concealing individual income, fabricating business, and switching the nature of income and underreported other taxes of 60 million yuan. A tax administrative penalty decision was made to impose a total of 1.341 billion yuan of overdue taxes and fines. Subsequently, by December 20, thousands of live streamers have made self-examination and remedial payments for overdue taxes and fines. The Global Times commented that it is hoped that the punishment of Weiya for tax evasion will lead to a wave of self-examination and self-correction of taxes for Internet celebrities.

Live streamers Zhu Chenhui and Huang Wei ranked third and first, respectively, in the sales ranking of Taobao's online live streaming. After Huang Wei's tax evasion being exposed, many live streamers actively pay back taxes. How to explain the taxpayer compliance behaviors of Zhu Chenhui, Lin Shanshan, Huang Wei, the other live streams' following tax repayment behaviors. A direct explanation is that the live streamers pay back taxes later actively to avoid punishments of tax evasion which could be detected in the future, and this is the deterrence effect of the tax authority's audit. Before this incident, the tax authority's collection and management of the online live streaming industry were loose to a large extent, as a result of which a large number of live streamers evaded tax. Another possible explanation based on the group psychology theory is that we consider the live streamers as a social group; therefore, a great quantity of tax evasion and tax repayment behaviors by the live streamers before and after the Hangzhou Taxation Bureau's audit can be regarded as a conformity behavior.

A deep understanding of the digital economy taxpayer compliance behavior is critical to formulating tax policies and reducing tax losses in the context of digital economy. However, few studies can be found in this area. The tax evasion case of live streamers in 2021 and the related research on taxpayer compliance show that the digital economy taxpayer compliance behavior could be affected by the deterrence of the tax authority and the conformity mentality of the taxpayer simultaneously (Traxler, 2010; Alm et al., 2017; Cyan et al., 2017; Solano-Garcia, 2017). Inspired by the case, we expect to delve into the digital economy taxpayer compliance behavior from these two dimensions. We develop a prospect theory model on tax compliance. In the model, we take the phenomenon that taxpayers will underestimate the small audit probability when the tax administration of digital economy is extremely loose into consideration, which is quite different from the model in the previous literature. Besides, a more important creative contribution is that we propose to determine the reference point based on a taxpayer's perceived compliance level (referring to the known average compliance level of other taxpayers), so that we can inspect what's the impact of other taxpayers' behaviors on the taxpayer compliance decision and further incorporate the conformity mentality of the taxpayer into our model. In order to make the model results easier to be understood and better fit the context of digital economy, we use the mixed methods of numerical simulation and case study.

The article is organized as follows. In Section Literature review and contributions, we briefly review the existing literature, explicate gaps in the existing research, and point out our contributions. In Section Model, we develop the model and analyze the deterrence effect and conformity effect in taxpayer compliance behavior. In Section Analysis of live streamers' tax compliance behaviors, we explain the tax compliance behavior of live streamers by using the results of model analysis. Section Conclusion and policy recommendations is conclusions and policy recommendations. The last section is the limitations of the article.

## Literature review and contributions

### Literature review

#### Digital economy tax research

At present, the research on digital economy tax can be classified into two paths: one is to analyze the causes of digital economy tax problems, and the other is to seek solutions to digital economy tax problems. Various new business models in the digital economy possess one common feature, that is, the value creation is decentralized and there is no need to rely on tangible entities, which results in three policy failures to the current tax legal system based on the industrial economy, including nexus determination method, data value measurement, and income characterization (Becker and Englisch, 2019). At the same time, the transaction characteristics of digitization, virtualization, and concealment in the digital economy make the information asymmetry between the tax authority and the taxpayer even worse, which seriously hinders the tax authority's collection and management. In coping with the digital economy tax problems, the organization for economic cooperation and development (OECD) has conducted ongoing research committed to find multilateral solutions and released the “two pillars” blueprint report in October 2020 (OECD, 2020a,b). Yet, a multilateral solution has not been figured out, but some countries such as the United Kingdom, France, India, Brazil, and others have issued unilateral digital services taxes as a stopgap (Cui, 2019; Vella, 2019). Based on the experiences from the practices of various countries and discussions of academic researchers, the specific collection and management schemes for digital economy taxation mainly include simplifying online tax registration, withholding tax through the network platform, information reporting of the network platform, using big data and blockchain technology to strengthen the collection and management (Choi and Hi-youl, 2004; Chriatia and Jih, 2006; Chase, 2020; Alm, 2021).

#### Tax compliance research based on prospect theory

Tax compliance research began in the 1970's (Andreoni et al., 1998; Slemrod, 2019), and the related theoretical models can be mainly summarized into three categories, mainly based on expected utility theory, game theory, and prospect theory. Allingham and Sandmo (1972) established an A-S model based on expected utility theory, regarding taxpayers as completely rational economic persons, and analyzed the impact of policy parameters, such as the tax rate, the audit probability, and the penalty rate on tax evasion. Yitzhaki (1974) made an important improvement to the A-S model, using the tax evasion amount of the taxpayer as the basis for calculating fines. The A-S model and Yitzaki's model, referred to as the A-S-Y model for short, are the basis for studying tax compliance issues. A basic conclusion of the A-S-Y model is that the taxpayers' compliance is out of the fear of tax evasion being discovered by tax authorities, and the increases in the audit probability and the penalty rate can make taxpayers more compliant, which means that the deterrence of tax authorities has an important impact on taxpayers' compliance behavior. However, some scholars argue that the conclusion of the A-S-Y model based on the assumption of a completely rational economic person could not fully explain the tax compliance behavior, and further they try to take the taxpayer's bounded rationality into the theoretical model, from which the tax compliance research based on prospect theory has achieved relatively rich results.

Prospect theory was first proposed by Kahneman and Tversky (1979), which is called the original prospect theory. Later, Tversky and Kahneman (1992) established a more complete “cumulative prospect theory” based on the rank-dependent utility theory proposed by Quiggin (1982). Prospect theory introduces psychological factors into the decision-making process, and its core idea can be specified into five points, namely, reference dependence, non-linear weighting of probabilities, decreasing sensitivity, loss aversion, and framing effect. Reference dependence means that the gain and loss are counted based on the reference point not the absolute value of the result. Non-linear weighting of probabilities means that the decision weight under uncertainty is not equal to the objective probability, and people usually overestimate the small probability and underestimate the moderate probability and high probability. Decreasing sensitivity means that the curve of the value function is concave in the gain area and convex in the loss area, thus forming an S-shaped curve which indicates that people are more sensitive to the difference closer to the reference point. Loss aversion means that losses are more salient for people compared with the same amount of gain. Framing effect means that people's preferences are influenced by the way a problem is described.

Yaniv (1999) is the early researcher who applied prospect theory to the tax evasion model and discussed the incentive effect of the tax advance payment system on tax compliance. In Yaniv's model, the reference point is the taxpayer's net income after deducting withholding tax from taxable income. Bernasconi and Zanardi (2004) comparatively analyzed the differences in tax compliance in the case of arbitrary reference point selection and proposed that the taxpayer's pretax income can be used as a reference point. Dhami and al-Nowaihi (2007) believed that the net income of the taxpayer after paying taxes according to the law should be used as a reference point. Their model successfully explained why most taxpayers choose to comply in the case of the low audit probability and penalty rate in reality and clearly indicated that tax evasion increases in the tax rate, which solved the Yitzaki mystery^1^.

#### Research on conformity effect of tax compliance

The research on the conformity effect of tax compliance is to analyze taxpayers' compliance decisions in the context of a social group, rather than to analyze the individual taxpayer's compliance behavior in isolation. The conformity behavior of tax compliance is related to social norms, and Wenzel (2005) regarded the social norms as the perceived frequency and acceptability of tax evasion among reference groups. Traxler (2010) established a tax compliance model with social norms, where the social norms are endogenously determined by the proportion of tax evaders in the whole society, so each taxpayer's compliance decision is not made independently but affected by other taxpayers. He proposed a policy of “belief management”, that is, taking steps to convince taxpayers a high level of overall social tax compliance would help reduce tax evasion. Solano-Garcia (2017) constructed a political competition model considering tax compliance fairness factors, in which the taxpayers' utility depends not only on consumption but also on the difference between their own tax evasion level and the perceived average tax evasion level. Moreover, based on natural experiments, Fellner et al. (2013) found that the households with lower tax compliance level in the community would increase tax compliance level after receiving the letter information which indicates that their neighbors had higher compliance levels. Alm et al. (2017) found through laboratory experiments that when taxpayers learn about their neighbors' tax compliance decision-making information, their tax compliance level may increase or decrease, because the neighbors' compliance level may be higher or lower, and there is a convergence effect in taxpayers compliance decisions. Through natural experiments, Cyan et al. (2017) found that the Pakistan Federal Revenue Agency's promotion of paragon compliant taxpayers through newspapers and televisions could improve taxpayers' perceived compliance levels and then further promote taxpayers' voluntary compliance.

### Contributions

Summarized from the literature on digital economy tax, scholars mainly analyze, from macro perspectives including the tax legal system and the tax administration mode, the mismatch between old taxation rules and new business models of digital economy, and then recommend relevant solutions. Few scholars have analyzed the compliance behavior of digital economy taxpayers from the micro perspective of taxpayers' compliance decision. Although the tax compliance problem has been discussed in plenty of literature and research, it has rarely been explored in the context of digital economy. So, this article aims to analyze the compliance behavior of digital economy taxpayers by establishing a tax compliance model based on prospect theory. Compared to previous research, this article is expected to make the following two contributions:

(1) To simultaneously investigate the impact of the deterrence effect and the conformity effect on taxpayer compliance behavior, this article develops a tax compliance model based on prospect theory, in which the decision reference point is determined by the taxpayer's perceived compliance level, which is the average compliance level of other taxpayers. The reference point is the taxpayer's net income after filing the tax return, which is all done based on the taxpayer's perceived compliance level. Using this reference point can effectively describe the taxpayer's conformity mentality.

The A-S-Y model based on expected utility theory focuses on the impact of the audit probability and the penalty rate on tax compliance. Since then, the deterrence effect has been the focus of various tax compliance models. However, the A-S-Y model does not take the interaction between taxpayers' behaviors into account. Although Traxler (2010) and Solano-Garcia (2017) consider the interaction between taxpayers' behaviors, they only extend a little bit of the A-S-Y model and still rely on the expected utility theory. In our research, the reference dependence of prospect theory facilitates the simultaneous analysis of the audit deterrence and the conformity mentality on the taxpayer compliance behavior. The crux is the selection of the reference point, but prospect theory itself does not provide the selection basis for tax compliance issues. From the existing literature on tax compliance based on prospect theory, there are two main reference points accepted by scholars. One is the taxpayer's taxable income after deducting advance tax (Yaniv, 1999); the other is the net income after tax according to the law (Dhami and al-Nowaihi, 2007). However, neither the reference point abovementioned captures the conformity mentality of the taxpayer. Unlike them, the reference point assumed in this article is based on the taxpayer's perceived compliance level. The reason is that a large number of studies on the conformity effect of taxpayer compliance, including theoretical and experimental research, clearly show that taxpayer compliance behavior will be affected by other taxpayers. Therefore, the reference point assumption in this article has a solid theoretical basis and also fits the reality, which reflects the behavioral characteristic of the taxpayer's reference tendency to the compliance levels of the others when making a tax compliance decision.

(2) Combined with the practical background of tax compliance and administration in digital economy, this article takes the situation into consideration that the taxpayer may underestimate the audit probability.

Research on experience decision found that people underestimate the probability of rare events, which are on contrary to the conclusions of prospect theory (Hertwig et al., 2004). Moreover, Kahneman and Tversky (1979) also pointed out that people may choose to ignore events with particularly small probability, and therefore, the estimation of probabilities close to zero is not stable. Camerer and Ho (1994) also compared the two situations of overestimating small probability and underestimating small probability when simulating the probability weight function. At present, since the tax authority has not yet comprehensively levied and managed in the digital economy, the audit probability of many digital economy business activities is much lower than that of the traditional ones, which result in that many digital economy taxpayers have neither personal experience of being audited nor the observations of their friends or peers being audited. So, it is more likely that these taxpayers will further underestimate rather than overestimate the audit probability. This point has a great impact on the compliance decisions of digital economy taxpayers, but it is not mentioned in the existing research on tax compliance based on prospect theory. This article will take this situation into consideration.

Through model derivation and numerical simulation, this article finds that the taxpayer compliance behavior mainly depends on the tax authority's enforcement effort (which is determined simultaneously by the audit probability and penalty rate), as well as the taxpayer's probability weight function and value function. The taxpayer compliance behavior can be divided into three scenarios, namely, complete tax non-compliance, partial tax compliance, and complete tax compliance. There are the deterrence effect and the conformity effect in the taxpayer compliance behavior, and both effects are affected by the tax authority's enforcement effort. The taxpayer chooses complete non-compliance because of such low enforcement effort of the tax authority, and in this case, neither deterrence effect nor conformity effect exists. If the tax authority appropriately strengthens enforcement, the taxpayer will choose partial compliance. At this time, the deterrence effect and the conformity effect coexist. If the tax authority enforces the law in a stricter way, the taxpayer will choose complete compliance because of the strong deterrence effect. This conclusion seems to be intuitive, but to our knowledge, the existing literature does not give such a conclusion. This article also analyzes the tax evasion case of live streamers and finds that the taxpayer compliance behavior of live streamers is consistent with the predictions of the model.

## Model

### Assumptions and notations

(1) The taxable income of a taxpayer *I* > 0 is exogenous. The taxpayer can choose to declare a certain amount *D* ∈ [0, *I*].
(2) The tax rate *t* and the audit probability *p* are fixed. It is assumed that the audit is perfect, that is, once the tax authority conducts an audit, the non-compliance of the taxpayer would be detected^2^. At this time, the taxpayer not only has to pay the evaded tax *t*(*I* − *D*) but also pay the fine π*t*(*I* − *D*) according to the evaded tax, where π is penalty rate.
(3) The taxpayer forms a judgment on the average compliance level of other taxpayers through news media reports and the tax compliance status information of their friends, that is, the perceived compliance level, denoted by α ∈ [0, 1].
(4) To simplify the model and facilitate the analysis, it is assumed that the tax evasion cost is zero.

### Formulation and solution

Let the final income of the taxpayer in the case of audit and non-audit by the tax authority be *I*_*a*_ and *I*_*n*_, respectively, then


(1) $$\begin{array}{l} I_{a}=I\left(1-t\right)-\pi t\left(I-D\right), \end{array}$$

(2) $$\begin{array}{l} I_{n}=I-tD. \end{array}$$

As mentioned above, based on the perceived compliance level, the taxpayer defines an income reference point *R* = *I*(1 − α*t*) where 0 ≤ α ≤ 1. If α = 1, the reference point is the net income of the taxpayer after paying taxes in accordance with the law, which is used by Dhami and al-Nowaihi (2007); if α = 0, the reference point is the taxpayer's pretax income. Because 0 ≤ α ≤ 1, so there is *I*(1 − *t*) ≤ *R* ≤ *I*. The taxpayer's gain or loss values with or without the tax authority's audit are as follows:


(3) $$\begin{array}{l} I_{ar}=I\left(1-t\right)-\pi t\left(I-D\right)-R, \end{array}$$

(4) $$\begin{array}{l} I_{nr}=I-tD-R. \end{array}$$

Since *I*(1 − *t*) ≤ *R* ≤ *I*, it is easy to know *I*_*ar*_ ≤ 0, that is, *I*_*ar*_ is always in the loss region. However, *I*_*nr*_ is either in gain region or in loss region, which is dependent on the values of *D* and *R*.

Let *I*_*nr*_ = *I* − *tD* − *R* = 0, there is $D=\frac{I-R}{t}=\alpha I$. If *D* ∈ [0, α*I*], *I*_*nr*_ ≥ 0 in the gain region; If *D* ∈ [α*I, I*], *I*_*nr*_ ≤ 0 in the loss region. The economic implication is that the taxpayer determines a declaration reference point α*I* based on the perceived compliance level α, and in the case of non-audit, if the declared income amount is lower than the declaration reference point, the taxpayer would feel gaining by retaining the more after-tax income; otherwise, the taxpayer would feel losing.

According to the cumulative prospect theory, the prospect of the taxpayer's compliance decision can be denoted by *f* = (*I*_*ar*_, *p*; *I*_*nr*_, 1 − *p*), which means that the probabilities of the final income of the taxpayer *I*_*ar*_ and *I*_*nr*_ are *p* and 1 − *p*, respectively. The prospect value of taxpayer is


(5) $$\begin{array}{l} V\left(f\right)=w\left(p\right)v\left(I_{ar}\right)+\left(1-w\left(p\right)\right)v\left(I_{nr}\right). \end{array}$$

In Equation (5), *w*(·) represents a non-linear increasing function that converts objective probability into the decision weight, and there are *w*(0) = 0, *w*(1) = 1, and *w*′(·) > 0. *v*(·) represents the value function of gain or loss, and there is *v*′(·) > 0; besides, *v*″(·) < 0 in the gain region, and *v*″(·) > 0 in the loss region.

Taking the first and second derivatives of *V*(*f*) with respect to *D*, there are


(6) $$\begin{array}{l} V^{\prime }\left(f\right)=t\left[w\left(p\right)\pi v^{\prime }\left(I_{ar}\right)-\text{(1}-w\left(p\right)\text{)}v^{\prime }\left(I_{nr}\right)\right], \end{array}$$

(7) $$\begin{array}{l} V^{\prime \prime }\left(f\right)=t^{2}\left[w\left(p\right)\pi^{2}v^{\prime \prime }\left(I_{ar}\right)+\text{(1}-w\left(p\right)\text{)}v^{\prime \prime }\left(I_{nr}\right)\right]. \end{array}$$

When *D* ∈ [0, α*I*], there are *I*_*ar*_ ≤ 0 and *I*_*nr*_ ≥ 0, so $v^{\prime \prime }\left(I_{ar}\right)>0$ and $v^{\prime \prime }\left(I_{nr}\right)<0$. At this time, the sign of *V*″(*f*) cannot be determined. The maximum point can be obtained at *D* = 0, *D* = α*I* or the point satisfying *V*′(*f*) = 0. When *D* ∈ [α*I, I*], there are *I*_*ar*_ ≤ 0 and *I*_*nr*_ ≤ 0, so *V*″(*f*) > 0. The maximum point is determined by comparing the values of *V*(*f*) at *D* = α*I* and *D* = *I*.

In summary, we discuss the maximum point of *V*(*f*) based on the following two cases.

(1) Case I

If there exists the interior point solution D^*^ satisfying $V_{D=D^{*}}^{\prime }\left(f\right)=0$ and $V_{D=D^{*}}^{\prime \prime }\left(f\right)>0$, or there is not an interior point solution in the domain of [0, α*I*], then we have max *V*(*f*) = max { *V*_*D* = 0_(*f*), *V*_*D* = α*I*_(*f*), *V*_*D* = *I*_(*f*)}.

According to Equations (3) and (4), we know that when *D* = 0, there are *I*_*ar*_ = (α − 1 − π)*tI* < 0 and *I*_*nr*_ = α*tI*≥0; when *D* = α*I*, there are *I*_*ar*_ = (α − 1)(1 + π)*tI* ≤ 0 and *I*_*nr*_ = 0; when *D* = *I*, there is *I*_*ar*_ = *I*_*nr*_ = (α − 1)*tI* ≤ 0.

Substitute the above results into Equation (5), we have


$$\begin{array}{l} V_{D=0}\left(f\right)=w\left(p\right)v\left(\left(\alpha -1-\pi \right)tI\right)+\left(1-w\left(p\right)\right)v\left(\alpha tI\right),\\ V_{D=\alpha I}\left(f\right)=w\left(p\right)v\left(\left(\alpha -1\right)\left(1+\pi \right)tI\right),\\ V_{D=I}\left(f\right)=v\left(\left(\alpha -1\right)tI\right). \end{array}$$

Comparing the taxpayer's prospect values at three different declared income levels mentioned above, we find that when the subjective audit probability *w*(*p*) or penalty rate π is so low that *V*_*D* = 0_(*f*) is greater than *V*_*D* = α*I*_(*f*) and *V*_*D* = *I*_(*f*), the taxpayer will declare zero income; when *w*(*p*) and π are high so that *V*_*D* = α*I*_(*f*) is greater than *V*_*D* = 0_(*f*) and *V*_*D* = *I*_(*f*), the taxpayer will declare α*I* that is the declaration reference point; when *w*(*p*) and π are high enough that *V*_*D* = *I*_(*f*) is greater than *V*_*D* = 0_(*f*) and *V*_*D* = α*I*_(*f*), the taxpayer will declare the total income.

In addition, the variety of the perceived compliance level will simultaneously change the prospect values of the three different declared income levels in the same direction. However, whether it will lead the taxpayer to make different choices or not is doubtful based on the above results. We will discuss this in depth through numerical simulations in Section Numerical simulations.

(2) Case II

If the existing interior point solution D^*^ satisfies $V_{D=D^{*}}^{\prime }\left(f\right)=0$ and $V_{D=D^{*}}^{\prime \prime }\left(f\right)\text{<}0$ in the domain of [0, α*I*], then we have $\max\;V\left(f\right)=\max\;\left\{\;V_{D=D^{*}}\left(f\right),V_{D=I}\left(f\right)\;\right\}\;$. The conditions of this situation are $V_{D=D^{*}}^{\prime }\left(f\right)>0$ and $V_{D=\alpha I}^{\prime \prime }\left(f\right)\text{<}0$, that is,


(8) $$\begin{array}{l} \frac{v^{\prime }\left(\alpha tI\right)}{v^{\prime }\left(\left(\alpha -1-\pi \right)tI\right)}<\frac{w\left(p\right)\pi }{\text{1}-w\left(p\right)}<\frac{v^{\prime }\left(0\right)}{v^{\prime }\left(\left(\alpha -1\right)\left(1+\pi \right)tI\right)}. \end{array}$$

According to Equation (8), we know that if the situation of Case II is hoped to occur, the values of *w*(*p*) and π cannot be too low or too high.

If the maximal value of *V*(*f*) is obtained at *D* = D^*^, then we can use the comparative static analysis method to investigate the impact on the taxpayer's declared income from changes of different factors, such as audit probability, penalty rate, tax rate, taxable income, and perceived compliance level. The analysis results are as follows:


(9) $$\begin{array}{l} \frac{\partial D}{\partial p}=-\frac{tw^{\prime }\left(p\right)\left[\pi v^{\prime }\left(I_{ar}\right)+v^{\prime }\left(I_{nr}\right)\right]}{V_{D=D^{*}}^{\prime \prime }\left(f\right)<0}. \end{array}$$

Since *w*′(·) > 0, *v*′(·) > 0 and $V_{D=D^{*}}^{\prime \prime }\left(f\right)\text{<}0$, then ∂*D*/∂*p* > 0 in Equation (9).


(10) $$\begin{array}{l} \frac{\partial D}{\partial \pi }=-\frac{tw\left(p\right)\left[v^{\prime }\left(I_{ar}\right)-\pi t\left(I-D\right)v^{\prime \prime }\left(I_{ar}\right)\right]}{V_{D=D^{*}}^{\prime \prime }\left(f\right)<0}. \end{array}$$

Since *v*′(·) > 0, $v^{\prime \prime }\left(I_{ar}\right)>0$, and $V_{D=D^{*}}^{\prime \prime }\left(f\right)\text{<}0$, then the sign of ∂*D*/∂π in Equation (10) cannot be determined.


(11) $$\begin{array}{l} \frac{\partial D}{\partial t}=-\frac{t\left[\left(1-w\left(p\right)\right)Dv^{\prime \prime }\left(I_{nr}\right)-w\left(p\right)\pi \left(\left(1+\pi \right)I-\pi D\right)v^{\prime \prime }\left(I_{ar}\right)\right]}{V_{D=D^{*}}^{\prime \prime }\left(f\right)<0}. \end{array}$$

Since $v^{\prime \prime }\left(I_{nr}\right)<0$, $v^{\prime \prime }\left(I_{ar}\right)>0$, and $V_{D=D^{*}}^{\prime \prime }\left(f\right)\text{<}0$, then ∂*D*/∂*t* < 0 in Equation (11).

The comparative static analysis of changes in taxpayer's taxable income is a bit more complicated. Drawing on the methods of Yitzhaki (1974) and Bernasconi and Zanardi (2004), let $r\left(\cdot \right)=-\frac{v^{\prime \prime }\left(\cdot \right)}{v^{\prime }\left(\cdot \right)}$ that refers to the definition of absolute risk aversion coefficient. Using Equations (6) and (7), there is


(12) $$\begin{array}{l} \frac{\partial D}{\partial I}=1-\frac{\left(1-t\right)\left[r\left(I_{ar}\right)-r\left(I_{nr}\right)\right]}{t\left[\pi r\left(I_{ar}\right)+r\left(I_{nr}\right)\right]}. \end{array}$$

Since $v^{\prime \prime }\left(I_{nr}\right)<0$ and $v^{\prime \prime }\left(I_{ar}\right)>0$, we have *r*(*I*_*nr*_) > 0 and *r*(*I*_*ar*_) < 0. According to Equation (7), we can get $V^{\prime \prime }\left(f\right)=-t^{2}w\left(p\right)\pi v^{\prime }\left(I_{ar}\right)\left[\pi r\left(I_{ar}\right)+r\left(I_{nr}\right)\right]$. Since*V*″(*f*) < 0, then π*r*(*I*_*ar*_)+*r*(*I*_*nr*_) > 0. Therefore, we know ∂*D*/∂*I* > 0 in Equation (12).

Besides, let *E* denote the tax evasion amount, and there is *E* = *I* − *D*, then


(13) $$\begin{array}{l} \frac{\partial E}{\partial I}=1-\frac{\partial D}{\partial I}=\frac{\left(1-t\right)\left[r\left(I_{ar}\right)-r\left(I_{nr}\right)\right]}{t\left[\pi r\left(I_{ar}\right)+r\left(I_{nr}\right)\right]}. \end{array}$$

Based on the above analysis, ∂*E*/∂*I* < 0 in Equation (13).


(14) $$\begin{array}{l} \frac{\partial D}{\partial \alpha }=-\frac{t^{2}I\left[w\left(p\right)\pi v^{\prime \prime }\left(I_{ar}\right)-\text{(1}-w\left(p\right)\text{)}v^{\prime \prime }\left(I_{nr}\right)\right]}{V_{D=D^{*}}^{\prime \prime }\left(f\right)}. \end{array}$$

Since $v^{\prime \prime }\left(I_{nr}\right)<0$, $v^{\prime \prime }\left(I_{ar}\right)>0$, and $V_{D=D^{*}}^{\prime \prime }\left(f\right)\text{<}0$, we have ∂*D*/∂α > 0 in Equation (14).

Based on all the above analysis, it can be seen that if the maximal value of *V*(*f*) is obtained at *D* = D^*^, the taxpayer's declared income increases in the audit probability, taxable income, and perceived compliance level but decreases in the tax rate. The change in tax evasion is on the contrary. The effect from the penalty rate on the taxpayer's declared income remains ambiguous.

In addition, we can analyze the impact of differences in the perceived compliance level on the distance between the taxpayer's compliance level and perceived compliance level:


(15) $$\begin{array}{l} \frac{\partial \left(\alpha -D/I\right)}{\partial \alpha }=\left(1-\frac{\partial D}{\partial \alpha }/I\right)=\frac{t^{2}w\left(p\right)\pi \left(1+\pi \right)v^{\prime \prime }\left(I_{ar}\right)}{V_{D=D^{*}}^{\prime \prime }\left(f\right)<0}. \end{array}$$

∂(α−*D*/*I*)/∂α < 0 in Equation (15) shows that with the improvement of the taxpayer's perceived compliance level, the taxpayer compliance level will be closer and closer to the perceived compliance level.

### Numerical simulations

According to the above analysis for two cases, if the reference point is determined based on the perceived compliance level, there are three possible situations for taxpayers' compliance behaviors, namely, complete tax non-compliance, partial tax compliance, and complete tax compliance. At the same time, it should be noted that, for partial tax compliance, the taxpayer compliance level does not exceed the perceived compliance level. What kind of situation will appear depends not only on the enforcement effort of the tax authority (determined by both the audit probability and the penalty rate) but also on the taxpayer's probability weight function and value function.

The numerical simulations will be conducted in the following to help understand the taxpayers' compliance behaviors in the three different situations more clearly. Drawing on the research of Dhami and al-Nowaihi (2007), the probability weight function proposed by Prelec (1998) is used in numerical simulations, and its form is


(16) $$\begin{array}{l} w\left(p\right)=e^{-{\left(-\ln\;p\right)}^{\gamma }},w\left(0\right)=0, \end{array}$$

where γ is the parameter of the probability weight function. If γ = 1, the probability weight is consistent with the objective probability; if 0 < γ < 1, the small probability is overestimated, and the medium and high probabilities are underestimated; if γ > 1, the small probability is underestimated, and the medium and high probabilities are overestimated^3^.

Adopting the value function proposed in Tversky and Kahneman (1992):


(17) $$\begin{array}{l} v(x)=\{\begin{array}{c} \;x^{\beta },\;\;\;\;\;\;\;\;\;\;x\geq 0\\ -\lambda {\left(-x\right)}^{\beta },x<0 \end{array} \end{array}$$

where λ > 1 and β < 1 are two parameters to, respectively, measure the loss aversion and risk aversion of decision makers.

The focus of this article is to analyze the influence of the tax authority's audit deterrence and the taxpayer's conformity mentality on the taxpayer's compliance behavior. Therefore, for the convenience of discussion, the values of some parameters are fixed in the simulations and set *I* = 100, *t* = 0.2, λ = 2.25, and β = 0.88^4^. For the setting of the audit probability, we refer to the audit documents of the Chinese tax authorities. The Chinese tax authorities conduct classified audits on taxpayers. The annual random audit probability for key tax source enterprises is about 20%; for non-key tax source enterprises, the annual random audit probability does not exceed 3%; for non-enterprise taxpayers, the annual random audit probability does not exceed 1%. Therefore, the audit probability *p* in the simulations can be set between 0 and 0.2, and the typical audit probability can be set to 0.01, 0.03, and 0.2, and the extremely low audit probability can be set to 0.001 in the case of very loose tax administration. According to the Chinese tax law, the penalty rate π is between 0.5 and 5. The perceived compliance level α can be set to 10 and 80%, respectively, representing the lower and higher tax compliance levels.

The numerical simulation results of the taxpayers' compliance behaviors are summarized into three scenarios, which are amplified as below.

#### Scenario 1: Complete tax non-compliance

**Figure 1:**
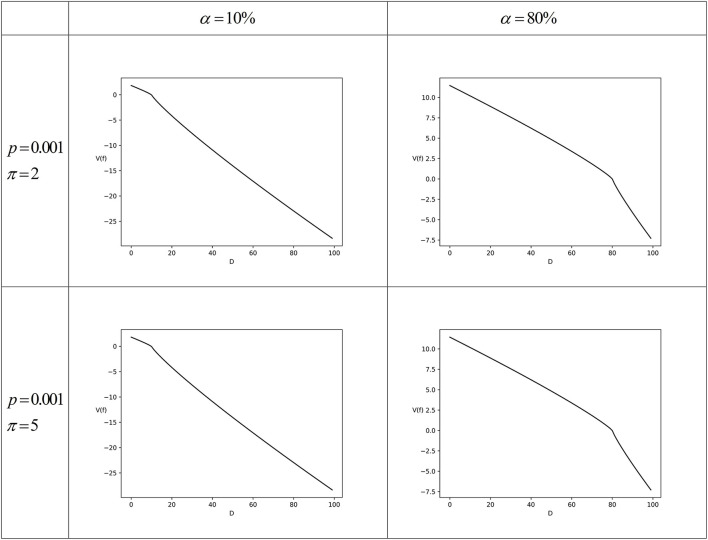
The numerical simulation for Scenario 1 (complete tax non-compliance).

Set *p* = 0.001, π ∈ {2, 5}, and γ = 1.1, and then *w*(0.001) = 0.0002 indicates that the taxpayer underestimates the audit probability. Figure 1 shows the numerical simulation results under different parameter combinations.

From Figure 1, it can be seen that the slopes of the taxpayer's prospect value function *V*(*f*) at *D* = α*I* are significantly different on the left and right sides. The reason has been described above, that is, when the taxpayer's declared income *D* < α*I*, the taxpayer's income relative to the reference point in the case of non-audit *I*_*nr*_ is located in the gain region; on the contrary, when *D* > α*I*, then *I*_*nr*_ is in the loss region. Due to loss aversion, the slope of the value function becomes larger when it switches from the gain region to the loss region. Therefore, according to Equation (6), the slopes of *V*(*f*) at *D* = α*I* from the left side to the right side will suddenly become smaller, which makes the curves appear steeper.

It should be clarified that although Figure 1 only shows the numerical simulation results for the perceived compliance level α = 10% and α = 80%, when α is modified to other values, the results still remain similar. That is, when other parameters remain unchanged, the value of α will not affect the taxpayer's decision of complete non-compliance.

The numerical simulation results shown in Figure 1 are consistent with the inferences in the previous theoretical analysis. The results indicate that when the taxpayer subjectively believes that the audit probability is very low, even though the fine on tax evasion will be charged 5 times of statutory maximum, the taxpayer still will not comply at all. The reason is that when taxpayers determine the reference point based on the perceived compliance level, they know that if they are not audited, the lower the tax compliance level compared with the perceived compliance level, the more they gain; but if they are audited, the lower the tax compliance level, the more the loss. Since the taxpayer who underestimates the audit probability believes that the probability of being detected for tax evasion is very low, it is the optimal decision to choose not to comply at all.

#### Scenario 2: Partial tax compliance

**Figure 2:**
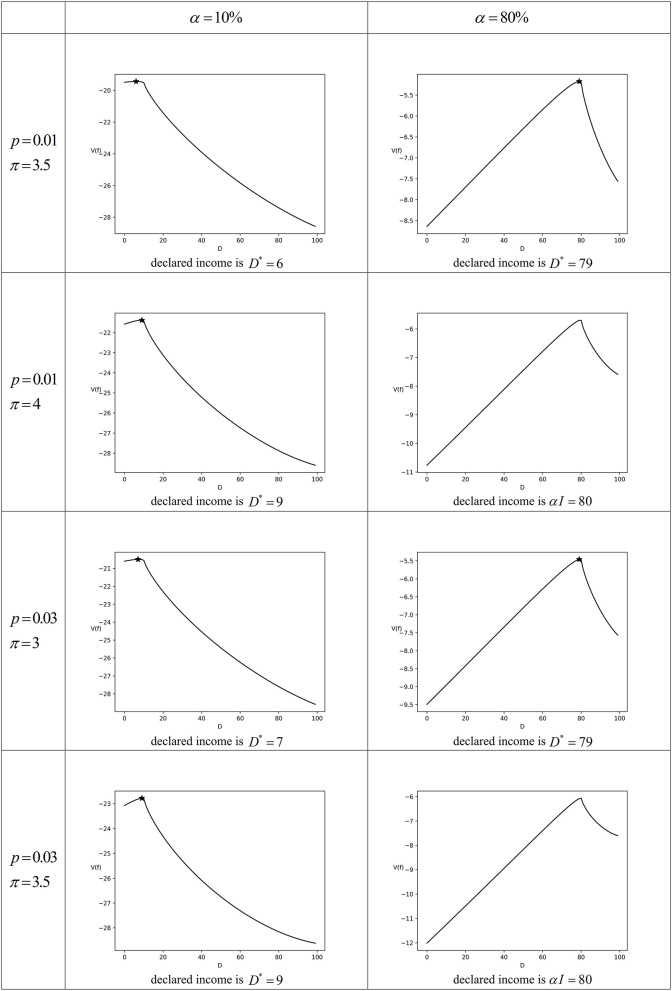
The numerical simulation for Scenario 2 (partial tax compliance).

Set the tax authorities' law enforcement parameter combinations (*p*, π) are (0.01, 3.5), (0.01, 4), (0.03, 3), and (0.03, 3.5), respectively. Set γ = 0.35^5^, and then *w*(0.01) = 0.181 and *w*(0.03) = 0.212, which mean that the taxpayer overestimates the audit probability. The numerical simulation results under different parameter combinations are shown in Figure 2, in which the asterisk represents the maximum interior point during the interval of [0, α*I*]. The numerical simulation results, when other parameters unchanged but only α changed, are similar to Figure 2.

The numerical simulation results in Figure 2 show that when the enforcement efforts of tax authorities are not very low or very high and meanwhile the taxpayer overestimates the audit probability, the taxpayer chooses the partial tax compliance. But the taxpayer compliance level does not exceed the perceived compliance level, because the taxpayer believes that there would be a certain loss related to other taxpayers if his/her own tax compliance level exceeds the perceived compliance level. Therefore, the taxpayer is more willing to take a risk to declare less income. However, the enforcement efforts of tax authorities have certain deterrence to the taxpayer, so the taxpayer will not entirely ignore the risk of paying an overdue tax and the penalty caused by complete tax non-compliance. Therefore, the taxpayer will choose to declare part of income, in which situation the tax compliance level is lower than or just equal to the perceived compliance level.

The numerical simulation results in Figure 2 are consistent with the aforementioned comparative static analysis, especially when the perceived compliance level increases, the taxpayer compliance level will be closer to the perceived compliance level. For example, for (*p*, π) = (0.01, 3.5), if α = 10%, the taxpayer compliance level is 6%, with a discrepancy of 4% from the perceived compliance level; and if α = 80%, the taxpayer compliance level is 79%, with a discrepancy of 1% from the perceived compliance level. For (*p*, π) = (0.03, 3), if α = 10%, the taxpayer compliance level is 7%, with a discrepancy of 3% from the perceived compliance level; if α = 80%, the taxpayer will follow the perceived compliance level as his/her compliance level, that is, the taxpayer fully imitates other taxpayers' behaviors with reference to social norms. In general, an increase in taxpayers' perceived compliance level will encourage them to comply more, which is consistent with Traxler (2010)'s tax compliance model with social norms.

It can also be inferred from Figure 2 that the increase in the audit probability will prompt the taxpayer to improve the compliance level. In addition, when the maximum value of *V*(*f*) is obtained at *D* = D^*^, although the impact of the penalty rate on the tax compliance level cannot be clearly judged by the comparative static analysis, the numerical simulation results show that an increase in the penalty rate will also prompt the taxpayer to improve the compliance level. It is consistent with the conclusion of the A-S-Y model based on the expected utility theory. However, it is a new finding that in Scenario 2, the taxpayer compliance level will not exceed the perceived compliance level, although the increases in the audit probability and penalty rate can encourage the taxpayer to be more compliant.

#### Scenario 3: Complete tax compliance

**Figure 3:**
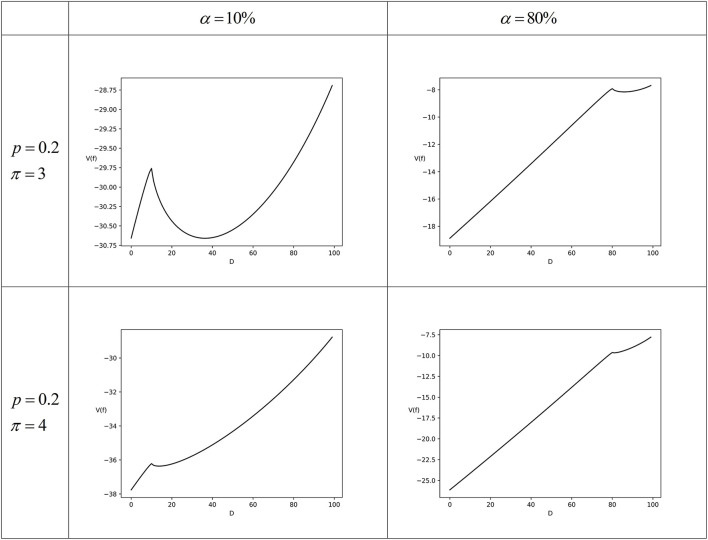
The numerical simulation for Scenario 3 (complete tax compliance).

Set *p* = 0.2, π ∈ {3, 4}, and γ = 0.35, then *w*(0.2) = 0.307 indicates that the taxpayer overestimates the audit probability. The numerical simulation results under different parameter combinations are shown in Figure 3.

The numerical simulation results in Figure 3 show that when the tax authorities' enforcement efforts are high enough and the taxpayer overestimates the audit probability, the tax authorities' enforcement deterrence is strong, and the taxpayer will choose to completely comply. When the value of α changed but other parameters remained unchanged, the numerical simulation results are similar to Figure 3.

### Analysis of deterrence effect and conformity effect

From three scenarios described above, it can be systematically summarized as follows:

(1) When the enforcement effort of the tax authority is very low and the taxpayer underestimates the audit probability, the taxpayer will choose complete non-compliance regardless of the perceived compliance level (i.e., Scenario 1). It indicates that the taxpayer's behavior is not influenced by other taxpayers under this situation, which means that there is no conformity effect when the tax authority's enforcement effort is very low. The reason for the taxpayer choosing complete non-compliance is that the tax authorities are very loose in their tax administrations, which results in no deterrence effect.
(2) When the enforcement effort of the tax authority is high and the taxpayer overestimates the audit probability, the taxpayer will choose complete compliance regardless of the perceived compliance level (i.e., Scenario 3). The taxpayer's behavior will also not be influenced by other taxpayers, and there is also no conformity effect, but the deterrence effect is very strong.
(3) When the enforcement effort of the tax authority is at a moderate level and the taxpayer overestimates the audit probability, the taxpayer chooses partial compliance (i.e., Scenario 2), and the higher the perceived compliance level, the higher the taxpayer compliance level and the closer it is to the perceived compliance level. For the fixed perceived compliance level, the taxpayer compliance level will increase and be closer to the perceived compliance level when the tax authority's enforcement effort is strengthened. Therefore, in Scenario 2, the deterrence effect and the conformity effect coexist in the taxpayer's compliance behavior, and the intensity of the deterrence effect is between that of Scenario 1 and Scenario 3.

**Figure 4:**
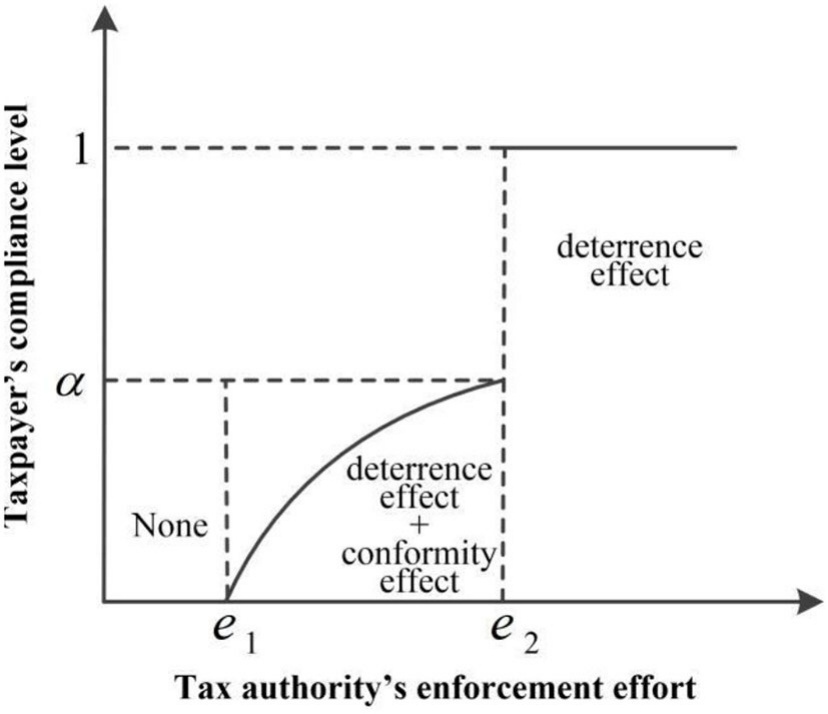
The relationship between the tax authority's enforcement effort and the taxpayer's compliance level.

Thus, the deterrence effect and conformity effect in the taxpayer's compliance behavior are affected by the tax authority's enforcement effort, and their relationships are described in Figure 4, where the horizontal axis represents the tax authority's enforcement effort denoted by *e* = *pπ*, and the vertical axis represents the taxpayer's compliance level. *e*_1_ and *e*_2_ are two critical points. When the enforcement effort is less than *e*_1_, the deterrence effect and the conformity effect do not exist, and the taxpayer does not comply at all; when the enforcement effort is greater than *e*_1_ and less than *e*_2_, the deterrence effect and the conformity effect both exist, and the taxpayer partially complies, but the compliance level will not exceed the perceived compliance level; when the enforcement effort is greater than *e*_2_, there is only a strong deterrence effect, and the taxpayer completely complies.

## Analysis of live streamers' tax compliance behaviors

### Income characteristics of live streamers

In order to study the tax compliance behaviors of live streamers, it is necessary to first analyze the income characteristics of live streamers, which mainly include the following three aspects:

(1) The sources of live streamers' incomes are diversified, including salary income, activity income, gift income, advertising income, and other incomes from the joint operation of online games, bringing goods, self-operated goods, and live streaming rooms.
(2) The nature of live streamers' incomes is complex, which determines the various forms of tax payment. The live streamer can sign a cooperation agreement with live streaming platforms or brokerage companies. If the labor contract is signed, the two parties are in a labor service relationship, and the live streaming income belongs to the streamer's labor income. The platform or brokerage company should withhold and remit the individual income tax of the labor income. If the employment contract is signed, the two parties are in an employment relationship. The live streamer receives the wage from the platform or the brokerage company, and the platform or the brokerage company should withhold and remit the individual income tax of the wage. The live streamer can also set up a sole proprietorship or an individual partnership. In this case, various incomes should be subject to individual income tax according to business income.
(3) The incomes of top live streamers and ordinary live streamers vary greatly. The monthly income of most ordinary live streamers is between 3,000 and 5,000 yuan; but the annual income of top live streamers can be as high as 100 million yuan, which can be referred to the published tax evasion data of Huang Wei and others.

### Analysis of tax compliance behavior of live streamers based on theoretical model

In the following part, we will use the tax evasion cases of Huang Wei and the others to analyze the tax compliance behaviors of live streamers, and at the same time verify the conclusions of our theoretical model.

The tax evasion cases of Huang Wei and the others in 2021 and the following events of active tax repayment by thousands of live streamers fully exposed the serious tax evasion problem in the online live streaming industry. At present, the Hangzhou Municipal Taxation Bureau has announced several tax evasion cases of the top live streamers with high incomes, so we firstly analyze the tax compliance behaviors of the top live streamers.

There are three main reasons for the top live streamers to evade taxes:

(1) There are loopholes in the current Chinese individual income tax law. Different tax rates are applied to the comprehensive income sources, including wages, salaries, and labor remuneration, and the income from business operation, equities, and dividends. The highest progressive tax rates are 45 and 35%, respectively. The top live streamers took advantage of loopholes in the tax law to convert the income from the nature of individual wages, salaries, and labor remuneration into business income by establishing a sole proprietorship or an individual partnership, and further significantly lower the tax rate through applying for approved collection method.
(2) The collection and management of tax authorities lag behind the development of the online live streaming industry. Before 2021, the top live streamers have not been audited as key tax sources.
(3) All taxpayers in the online live streaming industry have a weak awareness of tax compliance. Under the conditions of loopholes in the tax law and lax enforcement of tax authorities, when facing huge economic temptations, the top live streamers generally choose to evade taxes owing to their very low perceived compliance level.

The loopholes and improvements of the individual income tax law are not the focus of this article. We mainly analyze the tax compliance behavior of the top live streamers from the second and third reasons mentioned above. Although the online live streaming industry of China began in 2015, the tax authorities did not pay attention to the tax collection and management of this industry until 2021. In September 2021, the General Office of the State Administration of Taxation issued a notice, proposing that for live streamers who set up individual studios and enterprises, they should be guided to establish an accounting system in accordance with laws and regulations and use the method of auditing account to file taxes; the tax authorities should regularly perform tax risk assessments and further conduct one-to-one risk warnings to live streamers and urge rectifications with tax-related risks. It is obvious that the tax authorities' collection and management of the online live streaming industry lag behind. According to the tax authorities' classification audit principle, the annual random audit probability of non-key tax source enterprises shall not exceed 3%. Based on the previous theoretical analysis and the audit probability of 3%, the top live streamers will choose to partially comply, but the tax compliance level will not exceed the perceived compliance level. When the perceived compliance level is low, the tax compliance level of the top live streamers will be very low.

Although the tax evasion data of Huang Wei and other live steamers released by the Hangzhou Municipal Taxation Bureau is not in detail, it is still possible to roughly calculate Huang Wei's tax compliance level. There are two main ways for Huang Wei to evade taxes: one is to hide income, and the other is to fabricate business to convert the income nature. The tax evasion amount of Huang Wei's hidden income is 558 million yuan^6^. According to the progressive individual income tax rate of 45%, it is estimated that her hidden income is 1.24 billion yuan. The tax evasion amount of Huang Wei's fabricated business income is 116 million yuan. Since the live streamers belong to the modern service practitioners, the applicable taxable income rate is 10%, and the corresponding progressive individual income tax rate is 35%. The tax rate drops from 45 to 3.5% by converting the income nature, according to which it can be estimated that Huang Wei converted the income nature of 280 million yuan. In sum, it can be estimated that Huang Wei's whole income is about 1.52 billion yuan. She only declared 18.42% of her income, and certainly the tax rate, corresponding to the declared income, is very low. Although this is only an estimated result, it is enough to prove that the tax compliance behavior of the top live streamers is consistent with the theoretical results derived in this article. Due to their high incomes and social reputations, it is impossible for the top live streamers to completely ignore the tax administration. The tax audit still has a certain deterrence effect on them. Therefore, the top live streamers dare not be completely non-compliant. However, due to the lax tax enforcement, the deterrence effect is not strong. Under the circumstance of the low group tax compliance level, the top live streamers only declare a small part of their income driven by the conformity effect.

After Huang Wei's tax evasion was revealed, thousands of live streamers actively paid back their taxes. This phenomenon can also be explained by our model. The notice of the General Office of the State Administration of Taxation in September 2021 clearly requires one-to-one risk warnings, supervision, and rectification to the live streamers with tax-related risks, which shows that the tax authorities have begun to strengthen the administration of the online live streaming industry. For top live streamers, the tax authorities will regulate them as key tax sources with an audit probability of 20% or even higher. In addition, the Hangzhou Municipal Taxation Bureau successively detected tax evasions by Zhu Chenhui, Lin Shanshan, and Huang Wei and imposed fairly severe penalties. For example, for the 27 million yuan hidden income, Huang Wei did not actively pay back tax, the penalty was calculated on 109 million yuan, which is 4 times of the hidden income. Relevant news is widely disseminated and discussed in the major media, which definitely makes other top live streamers overestimate the audit probability, and know that if they do not actively pay back taxes, once caught, the penalty is very heavy. According to the theoretical analysis, the top live streamers will choose to actively pay back taxes. This belongs to the complete tax compliance as the result of a strong deterrence effect, but there is no conformity effect assumed at the beginning of this article. Although the Hangzhou Municipal Taxation Bureau has not stated the true income of the live streamers actively paying back taxes, it can be speculated that most of them belong to high-income group. For example, after Huang Wei's tax evasion case, Xie Qinhao, a live streamer whose income is up to 20 million in 2015, promised through Weibo that he would actively pay back all taxes.

Next, we analyze the tax evasion of ordinary live streamers. According to the “2020 China Online Performance (Live) Industry Development Report” released by the Online Performance (Live) Branch of the China Performance Industry Association, the total number of live streamer accounts has exceeded 130 million. It can be seen that there is a very large number of live streamers. Most live streamers are ordinary live streamers whose incomes are not high. They would not evade taxes by setting up a sole proprietorship or an individual partnership to convert the income nature like the top live streamers. But, for most of the ordinary live streamers, it is also impossible for the tax authorities to supervise them in the same way as the top live streamers. Based on the official audit principle, the random audit probability is no more than 1% for non-enterprise taxpayers each year. According to the theoretical model of this article, when the audit probability is very low, the ordinary live streamers will further underestimate the audit probability and thus completely fail to comply. So, for the ordinary live streamers, the most effective way to levy taxes is to withhold and remit taxes through live streaming platforms. However, when the tax authority's enforcement is not strict, the platform itself will not standardize the withholding and remitting taxes. For example, according to the data from the Beijing Municipal Taxation Bureau in 2017, from January to May of that year, there were only 5 live streaming platforms that receive individual income tax declaration from more than 1,000 individuals, 21 platforms receive individual income tax declaration from <10 individuals, and 6 platforms with zero declaration.

### Reasons for lax tax enforcement in online live streaming industry

The above analysis shows that an important reason for the serious tax evasion in the online live streaming industry is the lax tax enforcement and the following low deterrence effect. The reasons for the lax tax enforcement in the online live streaming industry can mainly be amplified from three aspects as follows:

First, the Chinese government has always been encouraging the development of digital economy and adopts an inclusive and prudential principle to regulate new business formats and new business models in the digital economy. For example, in July 2020, 13 departments including the National Development and Reform Commission, the Central Cyberspace Administration of China, and the Ministry of Industry and Information Technology jointly issued the “Opinions on Supporting the Healthy Development of New Business Formats and New Models, Activating the Consumer Market and Driving Employment Expansion.” The Opinions proposed is to accelerate the development of 15 new business formats and business models of the digital economy through 19 innovation supporting policies; and it is also firstly proposed to actively cultivate new individual economies, support self-employment, and encourage “sideline innovation.” Therefore, according to the inclusive and prudential principle, the tax authority will not strictly supervise the online live streaming industry in the early stage of its development.

Second, online live streaming is a new digital economic business model. The income sources of live streamers are diversified, and the live streaming business involves multiple agents, such as live streamers, platforms, brokerage companies, and individual enterprises. Moreover, there are some ambiguities existing in the definition of income source and the agent's withholding obligations in the current tax law. The tax authority needs a certain amount of time to study the business model before clarifying the taxation rules for the online live streaming industry. Prior to this, it was difficult for the tax authority to strictly supervise the online live streaming industry.

Third, the online live streaming industry has the characteristics of strong virtualization, high liquidity, and easy concealment. In the absence of platform data, there is a large degree of information asymmetry between the tax authority and the live streamers, and it is difficult to grasp the true income of the live streamers. Therefore, it is difficult to uncover live streamers who do not file taxes or verify the declaration of live streamers, which limit the tax authority's ability to regulate and supervise the online live streaming industry.

## Conclusion and policy recommendations

### Conclusion

Due to the mismatch between the traditional tax legal system and the new business model of digital economy, and the lagging tax administration, the digital economy is currently facing huge tax compliance problems. Taking the tax evasion of live streamers as the background case, this article establishes a tax compliance prospect theory model and analyzes the digital economy taxpayer compliance behavior from two dimensions including the tax authority's audit deterrence and the taxpayer's conformity mentality. In the model, we assume that the taxpayer estimate the average compliance level of other taxpayers comprehensively based on the information from various channels, and then a perceived compliance level is generated. Based on this perceived compliance level, the taxpayer further determines the income reference point. Such an income reference point makes it possible to incorporate taxpayers' conformity mentality into the model. As for the tax authority's audit deterrence, we take the tax compliance and administration both in the realistic context of the digital economy into consideration and include the situation that the taxpayer underestimates the small audit probability when the tax administration is extremely loose.

According to the model derivation and numerical simulation, we find that when the reference point is defined based on the perceived compliance level, there are three possible situations for the taxpayer compliance behavior: complete tax non-compliance, partial tax compliance, and complete tax compliance. The taxpayer's compliance behavior depends not only on the enforcement effort of the tax authority but also on the taxpayer's probability weight function and value function. There are two effects, namely, deterrence effect and conformity effect found in the taxpayer compliance behavior, and both effects are affected by the tax authority's enforcement effort. When the enforcement effort is low, the deterrence effect and the conformity effect do not exist, and the taxpayer does not comply at all. When the enforcement effort is moderate, the deterrence effect and the conformity effect coexist, the taxpayer partially complies, but the tax compliance level will not exceed the perceived compliance level, and an increase in the perceived compliance level will prompt the taxpayer to comply more. When the enforcement effort is high, there is only a strong deterrence effect, and the taxpayer completely complies.

This article theoretically explains the tax compliance behavior of live streamers and finds that the tax compliance behaviors of Huang Wei and other top live streamers are consistent with the prediction of the theoretical model. In the case of delayed tax administration and low perceived compliance level, the deterrence effect exists but is not strong, so the top live streamers will evade large amounts of taxes motivated by the conformity effect. However, the active tax repayment behavior of thousands of live streamers later after Huang Wei's tax evasion case is only the result of the strong deterrence effect but not related to the conformity effect.

### Policy recommendations

Based on the research conclusions, we propose the following three policy recommendations:

(1) Due to the platform-based characteristic of digital economy, the efficient tax administration for digital economy must rely on the cooperation method of “government + platform”. Through the cooperation with the platform, the tax authority can fully capture the income information of digital economy taxpayers, so as to implement classified management of digital economy taxpayers according to their income levels. For a small number of high-income digital economy taxpayers, the tax authority should include them into key tax sources and strengthen the relative audit. For most low- and middle-income digital economy taxpayers, the taxes should be collected through platform withholding and remitting to improve the tax collection efficiency because the tax authority lacks sufficient audit resources to manage these taxpayers.
(2) Make the best use of information technology methods, such as big data and artificial intelligence, to increase audit efficiency and audit probability. Based on the increased actual audit probability, various methods should be taken to raise the digital economy taxpayers' subjective audit probabilities. For example, the tax authority should often release digital economy tax policies and detected tax evasion cases on various news media and online platforms.
(3) Take various measures to enhance the perceived compliance levels of digital economy taxpayers. The tax authority should continue to strengthen the tax administration of high-income digital economy taxpayers to improve their compliance level and regularly announce typical cases of honest digital economy taxpayers on different news media and online platforms to picture them as social paragons. The tax authority can also capture the information of average tax payment level of different digital economy industries through big data analysis and send emails about the average industry tax payment level to the taxpayers whose tax payment are under the average, so as to improve their voluntary tax compliance.

## Limitations

There are still some limitations about this article. This article focuses on the impact of deterrence and conformity effects on digital economy taxpayer compliance behavior but does not consider the heterogeneity and decision-making dynamics of taxpayers. In the future, we will apply a multi-agent-based simulation method to formulate a model, which can include heterogeneous taxpayers' dynamic perceived compliance level and income reference point. In addition, since we are currently unable to obtain the micro-database of digital economy tax compliance, we cannot test the results of the model through statistical methods. The experimental methods could be used to further explore the questions of this article.

## Data availability statement

The original contributions presented in the study are included in the article/supplementary material, further inquiries can be directed to the corresponding author.

## Author contributions

PJ structured the whole paper, developed the model, analyzed live streamers' behavior based on the model, and wrote the first manuscript. GL reviewed the literature, conducted the numerical simulation, and verified the whole paper. WX collected the tax evasion case of live streamers and offered some good suggestions in Policy Recommendations. All authors contributed to the article and approved the submitted version.

## Funding

This study was sponsored by the National Social Science Fund of China under Grant 21BJY072.

## Conflict of interest

The authors declare that the research was conducted in the absence of any commercial or financial relationships that could be construed as a potential conflict of interest.

## Publisher's note

All claims expressed in this article are solely those of the authors and do not necessarily represent those of their affiliated organizations, or those of the publisher, the editors and the reviewers. Any product that may be evaluated in this article, or claim that may be made by its manufacturer, is not guaranteed or endorsed by the publisher.

## Footnotes

1   Yitzhaki (1974) shows that tax evasion decreases in the tax rate, but this conclusion does not conform with the intuition and contradicts the findings of many empirical studies.

2   If the audit is imperfect, the probability of the tax authority to find the tax evasion does not equal to the audit probability but equals the audit probability multiplied by the detection probability. In this case, *p* in the model can be interpreted as the probability of the tax authority to detect tax evasion. In addition, there are many taxpayers who have not registered for taxation in the digital economy. They will not declare any income, which means the declaring income is zero. In this case, *p* in the model is the probability that the tax authority screens out the taxpayer who has not been registered for taxation. Taking these cases into consideration does not change the conclusions of this article.

3   Prelec (1998) did not consider the situations where the small probability is underestimated and medium and high probabilities are overestimated, and therefore, the value of γ is defined between 0 and 1.

4   The values of λ and β are referred to Tversky and Kahneman (1992).

5   The larger the value of γ is, the smaller the degree of overestimating the small probability is. Referring to Dhami and al-Nowaihi (2007), we set γ = 0.35.

6   It includes 500 million yuan of hidden income tax evasion which is paid back, 31 million yuan of underpaid taxes that are voluntarily reported, and 27 million yuan of hidden income tax evasion which is not paid back.
